# Severe Hyperammonemic Encephalopathy Associated With Gastrointestinal Bleeding During Capecitabine and Oxaliplatin Chemotherapy in a Patient With Rectal Adenocarcinoma

**DOI:** 10.7759/cureus.109421

**Published:** 2026-05-22

**Authors:** Taiga Komatsu, Veevek Anand Agrawal

**Affiliations:** 1 Department of Internal Medicine, Mount Sinai Morningside/West, New York, USA; 2 Division of Hospital Medicine, Icahn School of Medicine at Mount Sinai, New York, USA

**Keywords:** capecitabine, fluoropyrimidine, gastrointestinal bleeding, hepatic encephalopathy, hyperammonemia, hyperammonemic encephalopathy, oxaliplatin, platinum

## Abstract

Hyperammonemic encephalopathy is a rare complication of fluoropyrimidine and platinum-based chemotherapy that can occur independently of hepatic dysfunction. While gastrointestinal bleeding is a well-established precipitant of hyperammonemia in cirrhosis, its role in patients treated with chemotherapy without liver disease has rarely been reported. An 87-year-old male with stage IIIA rectal adenocarcinoma, treated with capecitabine and oxaliplatin, presented with two episodes of acute altered mental status. The first, associated with mild melena and modest hemoglobin decline, resulted in moderate encephalopathy that resolved spontaneously. One week later, he presented with profound unresponsiveness requiring intubation. This episode was related to recurrent melena with a significant hemoglobin drop. Endoscopy revealed a Mallory-Weiss tear. Liver function tests and hepatic imaging were unremarkable. EEG showed diffuse slowing with triphasic waves, and ammonia was elevated at 128 µmol/L. Treatment with lactulose and rifaximin led to neurologic recovery and return to baseline. This case suggests a potential association between gastrointestinal bleeding and hyperammonemic encephalopathy in a non-cirrhotic patient receiving chemotherapy. Clinicians should consider hyperammonemia in the evaluation of acute altered mental status during or after these chemotherapy regimens, especially in patients with other possible predisposing factors such as gastrointestinal bleeding.

## Introduction

Hyperammonemic encephalopathy is a well-recognized neuropsychiatric complication of acute and chronic liver disease, most commonly cirrhosis, where impaired hepatic clearance leads to ammonia accumulation and cerebral dysfunction [1]. Ammonia is produced by gut bacteria and protein metabolism, and in cirrhosis, its buildup results in a spectrum of symptoms from mild altered mental status to coma [2]. Gastrointestinal bleeding is an established precipitating factor in cirrhotic patients, as increased protein load from blood breakdown further elevates ammonia levels [3].

In the absence of underlying liver disease, hyperammonemic encephalopathy is a rare but increasingly recognized complication of fluoropyrimidine and platinum-based chemotherapy, with an incidence of approximately 0.7% for fluoropyrimidines and even fewer cases for platinum agents [4,5]. The pathogenesis involves inhibition of the urea cycle and mitochondrial dysfunction, leading to ammonia and neurotoxic metabolite accumulation, and may occur independently of hepatic dysfunction [4,6,7]. Most cases present with mild to moderate symptoms and resolve rapidly after discontinuation of chemotherapy and supportive care [5,8]. Known risk factors include advanced age, sarcopenia, renal impairment, dehydration, and infection [5,9].

Although gastrointestinal bleeding is a well-recognized precipitant of hyperammonemic encephalopathy in cirrhosis, its contribution in patients undergoing chemotherapy without underlying liver dysfunction remains unclear [3]. In cirrhotic patients, intraluminal blood provides a large protein substrate for ammonia-producing gut bacteria, and portal systemic shunting bypasses hepatic detoxification, resulting in a rapid rise in systemic ammonia levels [3]. In non-cirrhotic patients receiving chemotherapy, a similar increase in gut-derived ammonia from intraluminal blood may overwhelm a urea cycle already compromised by chemotherapy-induced metabolic inhibition, thereby precipitating hyperammonemia even in the absence of liver disease. To our knowledge, this potential synergistic mechanism has not been previously explored in the literature. This case reports severe hyperammonemic encephalopathy presenting with profound neurologic deterioration, requiring intubation and intensive care, in a patient receiving chemotherapy without liver dysfunction. We hypothesize that gastrointestinal bleeding served as a precipitating factor by providing an additional ammonia load that exceeded the detoxification capacity of the urea cycle, already impaired by chemotherapy, and we present this case to highlight this potential and clinically important association.

## Case presentation

An 87-year-old male recently diagnosed with stage IIIA rectal adenocarcinoma experienced two episodes of acute altered mental status during the course of chemotherapy. Several months before these events, he had completed chemoradiation therapy with capecitabine and subsequently transitioned to combination chemotherapy with capecitabine and oxaliplatin. He received four cycles in total, with the last cycle approximately four weeks before the neurologic deterioration. At baseline, he was fully independent in activities of daily living and was consistently alert and oriented.

Approximately two weeks before the latter hospitalization, he developed sudden aphasia and was found to be unresponsive. On arrival, he was nonverbal, not following commands, and intermittently withdrawing to tactile stimuli. Laboratory evaluation revealed a white blood cell (WBC) count of 4,100/µL, hemoglobin of 10.7 g/dL, and normal electrolyte levels. Stroke workup, including computed tomography (CT) of the head and magnetic resonance imaging (MRI) of the brain, demonstrated no intracranial hemorrhage or acute ischemia. Although cerebrospinal fluid (CSF) analysis and meningitis/encephalitis polymerase chain reaction (PCR) testing were negative, empiric therapy for possible meningoencephalitis was initiated with broad-spectrum antibiotics and antiviral therapy. During this first admission, he developed one episode of melena, accompanied by hemoglobin decline to 7.9 g/dL, though he remained hemodynamically stable. Upper endoscopy was performed, but it did not identify a source of bleeding. Hemoglobin remained stable after the endoscopy, and transfusion was not required. Over several days, his mental status steadily improved. He became oriented, appropriately interactive, and able to converse normally. He was discharged to a rehabilitation facility with full return to his cognitive baseline.

One week after the discharge to the rehabilitation facility, he developed a second, more severe episode of altered mental status. He initially reported feeling unwell but rapidly became nonverbal and unable to follow commands. Emergency medical services found him responsive only to tactile stimuli. On admission, he was tachycardic, and initial laboratory evaluation revealed a WBC count of 6,400/µL, hemoglobin of 6.0 g/dL, platelet count of 345,000/µL, blood urea nitrogen (BUN) of 23 mg/dL, creatinine of 0.99 mg/dL, aspartate aminotransferase (AST) of 30 U/L, alanine aminotransferase (ALT) of 14 U/L, alkaline phosphatase (ALP) of 52 U/L, total bilirubin of 0.8 mg/dL, lactate of 7.2 mmol/L, prothrombin time-international normalized ratio (PT-INR) of 1.9, and vitamin B12 of 638 pg/mL, with normal electrolyte levels. CT of the head again showed no acute pathology. CT abdomen demonstrated an unremarkable liver and bile ducts without ascites (Figure 1).

**Figure 1:**
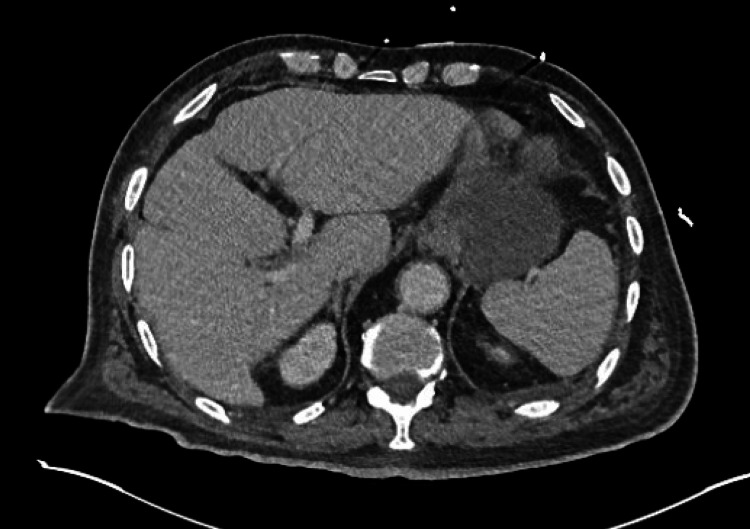
Computed tomography (CT) of the abdomen obtained during the second admission. The CT scan demonstrated a normal liver without ascites.

Given his severely altered mental status and aspiration risk, he was intubated for airway protection and admitted to the intensive care unit. Because of tachycardia and markedly elevated lactate, he was started on broad-spectrum antibiotics for suspected sepsis. Levetiracetam was also initiated for possible seizure; however, electroencephalography (EEG) demonstrated diffuse slowing with triphasic waves, without epileptiform discharges. His hospital course was complicated by recurrent episodes of melena, with hemoglobin eventually dropping to 4.3 g/dL. He received a total of five units of packed red blood cells. Upper endoscopy revealed a Mallory-Weiss tear requiring endoscopic clipping. Further evaluation during these episodes of melena demonstrated a markedly elevated ammonia level of 128 µmol/L, prompting initiation of lactulose and rifaximin. Over the following days, his ammonia levels decreased with progressive improvement in his mental status. He was successfully extubated on hospital day 5. No seizure-like activity was observed during the admission, and levetiracetam was discontinued on day 6. Blood cultures came back negative, and antibiotics were completed on day 7. He continued to improve and ultimately returned to his baseline level of alertness and orientation. He was discharged to the rehabilitation facility on day 15 (Figure 2).

**Figure 2:**
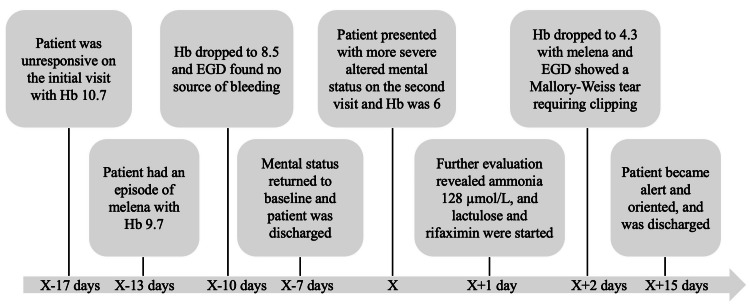
Timeline of the two hospitalizations. Image credit: Authors. EGD, esophagogastroduodenoscopy; Hb, hemoglobin (g/dL)

## Discussion

Hyperammonemic encephalopathy is a rare but clinically significant complication of chemotherapy, particularly with fluoropyrimidines and platinum-based agents. Although the overall incidence is low, it is crucial to include hyperammonemia in the differential diagnosis for patients presenting with acute altered mental status during or after chemotherapy, regardless of hepatic function [10-12]. This condition is likely underdiagnosed due to low clinical suspicion and under-testing, especially with oral agents such as capecitabine [10,12].

Chemotherapy-induced hyperammonemic encephalopathy is most commonly associated with fluoropyrimidines such as capecitabine and 5-FU, and, less frequently, platinum agents such as oxaliplatin [4,10]. The pathogenesis involves inhibition of the Krebs cycle by fluoroacetate, a metabolite of capecitabine and 5-FU, resulting in decreased ATP production and impaired urea cycle function [4,6,7,10]. Oxaliplatin and other agents may further disrupt mitochondrial metabolism, contributing to increased ammonia accumulation [4]. In susceptible individuals, including those with occult urea cycle defects or acquired mitochondrial dysfunction, ammonia clearance is further compromised, leading to neurotoxicity [13-15].

Hyperammonemic encephalopathy associated with chemotherapy typically presents as acute altered mental status, ranging from mild confusion and lethargy to more severe manifestations such as seizures and coma. While most reported cases are mild to moderate in severity, this case was exceptionally severe, manifesting as profound neurologic deterioration necessitating intubation and intensive care. The onset of symptoms varies, ranging from days to months after chemotherapy [5,8]. Laboratory findings usually reveal markedly elevated plasma ammonia levels, frequently in the absence of hepatic dysfunction [9]. Imaging is generally unremarkable, and EEG may show diffuse slowing or triphasic waves, consistent with metabolic encephalopathy [1].

Emerging evidence suggests that combination chemotherapy with capecitabine and oxaliplatin may further increase the risk of hyperammonemia, likely due to additive or synergistic inhibition of ammonia metabolism. Literature reviews suggest that this combination can multiply the risk of hyperammonemic encephalopathy, even in the absence of predisposing factors or impaired liver function [4].

Multiple risk factors have been identified that predispose patients without underlying liver dysfunction to hyperammonemic encephalopathy associated with chemotherapy. Renal dysfunction is a major contributor, as reduced glomerular filtration impairs ammonia clearance and increases susceptibility, especially during regimens like FOLFOX [9,16]. Dehydration and infection are also frequently implicated, with both conditions promoting azotemia and catabolic stress, further elevating ammonia levels [5,9]. Constipation can slow ammonia excretion, while sarcopenia and muscle loss reduce peripheral ammonia detoxification capacity [5]. Genetic predispositions, including occult urea cycle defects and acquired deficiencies in enzymes such as carbamoyl phosphate synthetase, have been described in patients who develop severe or refractory hyperammonemia during chemotherapy [13]. High tumor burden, total parenteral nutrition, and exposure to other drugs such as valproic acid are additional risk factors, particularly in critically ill or malnourished patients [17]. In cirrhotic patients, the relationship between gastrointestinal bleeding and hyperammonemia is well-established and multifactorial. When blood enters the gastrointestinal tract, hemoglobin is broken down by colonic bacteria and mucosal enzymes, releasing substantial quantities of ammonia that are absorbed into the portal circulation. In cirrhosis, this ammonia burden is amplified by impaired hepatic clearance and portosystemic shunting that diverts ammonia-rich portal blood away from the liver [1,3,18].

This case represents a potentially novel finding that gastrointestinal bleeding may precipitate hyperammonemic encephalopathy in patients receiving chemotherapy without underlying liver dysfunction. The pathophysiologic mechanism likely involves synergistic interaction between chemotherapy-induced impairment of ammonia metabolism and the increased nitrogen load from blood breakdown [3,4,6,7]. In the setting of this chemotherapy-induced metabolic derangement, the additional ammonia burden from gastrointestinal bleeding can overwhelm compensatory mechanisms and precipitate acute encephalopathy. The temporal relationship observed in this case is particularly striking. During the first admission, intermittent melena with mildly decreased hemoglobin was associated with only mild mental status changes. In contrast, the second admission was characterized by recurrent melena with significantly worsening anemia, indicating substantial gastrointestinal bleeding that precipitated more severe hyperammonemia and profound encephalopathy. While hyperammonemia is a recognized rare complication of fluoropyrimidine and platinum-based chemotherapy, established risk factors include renal impairment, dehydration, infection, and sarcopenia [4,5,8,9]. The addition of gastrointestinal bleeding as a precipitating factor in non-cirrhotic chemotherapy patients has not been clearly documented in the medical literature and warrants further investigation. Clinicians should maintain high suspicion for hyperammonemia in patients receiving fluoropyrimidine-platinum combinations who develop gastrointestinal bleeding, even in the absence of liver dysfunction. Prompt measurement of plasma ammonia levels should be considered in any patient with unexplained neurological symptoms during chemotherapy, as early recognition and intervention are critical to prevent irreversible neurological damage and death [5,8].

## Conclusions

Severe hyperammonemic encephalopathy is a rare but potentially life-threatening complication of fluoropyrimidines and platinum-based chemotherapy. This case highlights the importance of considering hyperammonemia in the evaluation of acute altered mental status during or after these chemotherapy regimens, especially in patients with other possible predisposing factors such as gastrointestinal bleeding.
